# Capturing clinically actionable copy number alterations in Wilms tumor using nanopore sequencing

**DOI:** 10.21203/rs.3.rs-8206667/v1

**Published:** 2026-01-25

**Authors:** Larissa V. Furtado, Carolyn Jablonowski, Pandurang Kolekar, Teresa Santiago, Christopher L. Morton, Allison Woolard, Andrew M. Davidoff, Xiaotu Ma, Andrew J. Murphy

**Affiliations:** Department of Pathology, St. Jude Children’s Research Hospital; Department of Surgery, St. Jude Children’s Research Hospital, Memphis; Department of Computational Biology, St. Jude Children’s Research Hospital; Department of Pathology, St. Jude Children’s Research Hospital; Department of Surgery, St. Jude Children’s Research Hospital; Department of Surgery, St. Jude Children’s Research Hospital; Department of Surgery, St. Jude Children’s Research Hospital; Division of Pediatric Surgery, Department of Surgery, University of Tennessee Health Science Center; Department of Computational Biology, St. Jude Children’s Research Hospital; Department of Surgery, St. Jude Children’s Research Hospital; Division of Pediatric Surgery, Department of Surgery, University of Tennessee Health Science Center

**Keywords:** Wilms tumor, nanopore, ONT, sequencing, copy number alterations, methylation

## Abstract

**Background:**

Copy number alterations (CNVs) involving chromosomes 1p, 1q, 16q, and 11p15 are key genomic markers used in the risk stratification of Wilms tumor (WT). These CNVs, when considered alongside disease stage and other clinical features, are associated with an increased risk of relapse. Accordingly, testing for these changes is recommended to guide treatment choices in children with favorable histology disease. Current methods for detecting segmental CNVs in WT, including single nucleotide polymorphism (SNP) arrays and short-read sequencing, require prolonged turnaround times, high cost, and do not capture loss of imprinting (LOI) at 11p15, a key adverse predictor for patients who would otherwise have very low risk disease. In this study, we assessed the feasibility of utilizing nanopore sequencing for CNV and 11p15 LOI analysis in WT.

**Methods:**

We performed whole-genome sequencing (WGS) using the MinION platform on 15 patient-derived WT xenografts previously characterized by whole exome sequencing (WES) and multiplex ligation-dependent probe amplification (MLPA), applying adaptive sampling in a subset to enrich for clinically relevant regions. End-to-end sequencing analysis was completed within 4 days.

**Results:**

Despite low sequencing depth (average 6.7x, WGS; 8.5x, adaptive sampling sequencing), nanopore WGS detected 94% (16/17) of the CNVs that are clinically relevant in WT. No false-positive findings were observed. Adaptive sampling showed high concordance with WGS for CNV detection and allowed sample multiplexing. However, the sequencing depth obtained by nanopore WGS and adaptive sampling was insufficient for assessment of methylation status at the Imprinting Control Regions (ICR)1 and ICR2 at 11p15.

**Conclusions:**

This study demonstrates the feasibility of using nanopore sequencing for the assessment of clinically relevant CNVs in WT and highlights the potential of this technology for the integrated evaluation of copy number variation and 11p15 methylation status with a much shorter turnaround time and lower cost than other conventional methods for CNV testing. Successful clinical integration will require higher throughput nanopore sequencing platforms, as indicated by the current findings.

## BACKGROUND

Wilms tumor (WT) is the most common kidney cancer of childhood^1^. The goals of treatment are to maximize cure while appropriately risk stratifying patients to minimize long-term toxicity of therapy by selecting intensified treatment for patients with a high risk of relapse and minimizing treatment for patients with excellent prognosis. In combination with disease stage, histology findings, and additional clinical features, the presence of specific somatic molecular alterations, such as loss of heterozygosity (LOH) of 1p and 16q, 11p15.5, and 1q gain is associated with an increased risk of relapse after initial therapy for patients diagnosed with WT^2–6^. Therefore, timely cytogenetic and molecular testing for 1q gain and/or LOH of 1p, 16q, and 11p15.5 is recommended for all children with newly diagnosed favorable histology WT since it would impact therapy decisions^7^.

In the current clinical practice, these segmental copy number variants (CNVs) are typically evaluated using single nucleotide polymorphism (SNP) array or short-read sequencing methods (e.g., Illumina sequencers). However, the cost of these tests is high (per sample cost around US$ 2,300 for the SNP array recommended by the Children’s Oncology Group for WT CNV testing and US$2,600 for the Illumina WGS sequencing performed in our institution for tumor-only CNV assessment), and the turnaround time is approximately 2–4 weeks. In addition, neither short-read sequencing nor the current SNP array testing recommended by the Children’s Oncology Group (COG) assesses methylation status or loss of imprinting (LOI) at the 11p15.5 locus. Methylation abnormalities at this site have been identified as adverse prognostic markers in young patients with stage I disease^66^.

Nanopore sequencing [Oxford Nanopore Technologies (ONT), Oxford, United Kingdom] offers multiple advantages over conventional methods for CNV testing. It provides long- and short-read sequencing information with quick turnaround times (2–5 days), simple sample preparation (direct, PCR-free sequencing of native DNA), genome-wide or targeted methylation profiling data for differential methylation analysis without the need for bisulfite conversion or additional wet laboratory steps, real-time analysis, and lower cost (approximately US$800 per sample with the MinION platform). In addition, targeted analysis of clinically relevant genomic regions can be carried out with adaptive sampling^8^ without the need for the design and optimization of primers or additional reagents. Adaptive sampling may be particularly advantageous for WT CNV testing, where a limited number of chromosome arm-level CNVs are of clinical interest, and due to the flexibility to include other alterations that may be clinically actionable in the future, such as *TP53* mutation or 17p13 LOH^9, 10^.Ultra-low pass nanopore whole genome sequencing has demonstrated promising results for the detection of arm-level alterations and high-level focal alterations in central nervous system tumor samples^11, 12^and in cell-free tumor DNA in cerebrospinal fluid of patients with gliomas^13^ and lung cancer^14^.

In this study, we assessed the feasibility of utilizing nanopore (ONT) sequencing for targeted CNV and 11p15 LOI analysis in WT.

## METHODS

### Study Samples

The study included 15 WT patient-derived xenograft (PDX) samples which had their copy number profile previously characterized by whole exome sequencing (WES) and multiplex ligation-dependent probe amplification (MLPA)^15^ (Table 1). The samples selected for this proof-of-concept study harbored CNVs that are clinically relevant in WT (i.e., 1p deletion, 1q gain, 11p15 deletion, and 16q deletion) as well as other CNV changes to broadly evaluate specificity and performance of the assay for detection of a wide spectrum of CNVs. Samples KT-22, KT-35, and KT-45 did not have any copy number changes relevant for WT risk stratification detected previously, therefore, serving as internal negative controls for this analysis. All samples had greater than 70% tumor purity.

### DNA isolation

DNA was isolated from frozen PDX samples using DNeasy blood and tissue kit (Qiagen, Germantown, MD) according to the manufacturer’s protocol. DNA concentration was determined by Qubit 4 Fluorometer (Thermo Fisher Scientific, Waltham, MA) and sample purity was assessed by Nanodrop One (Thermo Fisher Scientific).

### Library Preparation and Nanopore Sequencing

Following extraction and DNA quality control, fragmentation was achieved using a g-TUBE (Covaris) aiming for ~ 9kbp fragment length. Fragment size was assessed on a 4150 TapeStation system (Agilent Biotechnologies, Santa Clara, CA) using genomic screentape. Sequencing libraries for individual whole genome analysis were generated using the SQK-LSK114 ligation kit from Oxford Nanopore Technologies (ONT), according to the manufacturer’s instructions. Ligation and barcoding for multiplexed adaptive sampling were generated using the Ligation sequencing gDNA Barcoding Kit 24 V14 (SQK-NBD114.24) from ONT. For DNA sequencing, sample libraries were individually loaded onto MinION Mk1B flow cells (CAT No. FLO-MIN114) following ONT’s standard instructions for 72 hours. All samples underwent whole genome sequencing (WGS).

**Figure 1.**
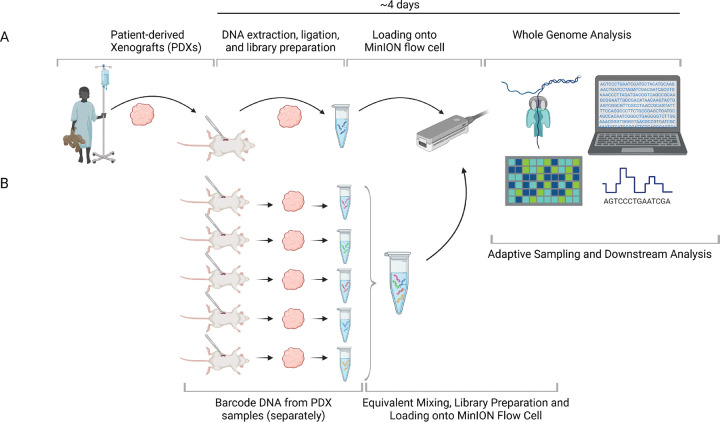
Overview of the experimental and analytical workflow of the study. (**A**) Schematic representation of the ONT WGS workflow beginning with nucleic acid extraction from patient-derived xenograft (PDX) specimens, followed by library preparation, sequencing on the MinION platform, and computational analysis. (**B**) DNA from the 5 PDX samples subjected to adaptive sampling was individually barcoded during library preparation for a multiplexed sequencing run performed using the MinION system. Created with BioRender.com

Sequencing and data acquisition were performed using ONT’s MinKNOW v25.05.12 and Dorado v 7.6.8 for raw signal values using a MacBook Pro with Apple M3 Pro chip 12-core CPU and 18-core GPU, Space Black, 36GB, 4TB Solid-State Drive (SSD), 140 W USB-C Power Adapter (Fig. 1).

### Adaptive Sampling

In addition to WGS, we performed one adaptive sampling sequencing run including 5 samples (KT-22, KT-25, KT-47, KT-55, and KT-60, Table 1) (Fig. 1). These samples were selected for this analysis because in addition to harboring CNVs that are clinically relevant in WT, some had methylation changes in 11p15 (KT-25, KT-47, and KT-60), as well as copy number changes involving the *WT1* and *AMER1* genes, which are frequently altered in WT (KT-22, KT-47, KT-55, and KT-60). The objective of this analysis was to determine the extent to which targeted sequencing enhances coverage depth and resolution of methylation status. Adaptative sampling was used to enrich for a) sequences of chromosomes 1, 11, 16, and 17, b) 45 genes that are commonly mutated genes in WT, c) genomic regions that are known not to be typically affected by copy number alterations in this tumor type (copy neutral internal controls), and d) imprinting control regions (ICR) around *H19/IGF2*^10, 16^. The targeted regions encompassed approximately 506 Mbp, which represents ~ 15.8% of the human genome. Even though the focus of the current study is on the detection of CNVs that are clinically relevant in WT, we decided to include other genomic regions that are associated with this tumor to allow for future analysis. The Browser Extensible Data (BED) file was checked using ONT’s Bed Bugs (https://epi2me.nanoporetech.com/bed-bugs/, last accessed on 06/20/2025), and a 10kbp buffer was added to each area of interest prior to sequencing.

### Bioinformatic Analyses

The base calling of raw reads generated by the ONT platform was performed with dorado v7.6.8 (https://github.com/nanoporetech/dorado) using DNA base calling model dna_r10.4.1_e8.2_400bps_hac@v4.3.0. All the reads that passed the base calling model were mapped against the reference human genome GRCh38 (hg38) assembly using minimap2 v2.24^16^ and depth of coverage was determined using mosdepth v0.3.3^17^.

CNVs for ONT WGS long read data were evaluated using QDNAseq v1.34^18^. Modified bases, such as methylation of CpG sites, were called out and summarized using modkit v0.3.3 (https://github.com/nanoporetech/modkit). The nextflow based wf-human-variation pipeline v2.6.0 (https://github.com/epi2me-labs/wf-human-variation) was used to execute these steps.

CNV analysis for short-read WES data that were previously generated for the samples included in this study^15^ was carried out using CNVKit v0.9.10^19^. The arm-level CNVs detected using ONT WGS long read data were compared to those detected from short-read WES using their log2 scores visualized using CNVKit heatmap. A cutoff of absolute log2 ratio of 0.4 (corresponding to gain of 0.6 copies) is used in this work.

Methylation profiles of ICR around *H19/IGF2* (ICR1) and *KCNQ1OT1* (ICR2) for the samples reported to show gain in methylation by previous methylation-sensitive MLPA analysis (Table 1) were compared with commercially available male (CAT No. G1471, Promega, Fitchburg, WI) and female (CAT No. G1521, Promega, Fitchburg, WI) control DNA samples. Methylartist v1.4.0^20^ and figeno v1.8.1^21^ were used to visualize methylation profiles in ICR regions. Mann-Whitney U test was used to assess for statistically significant difference in methylation between different groups of samples.

## RESULTS

### Quality Assessment of ONT Sequencing Data

All samples (n = 15) sequenced using ONT WGS had genome wide average depth of coverage of at least 5x (range: 5–8x, average: 6.7x, median: 7x). The statistics related to the number, length, N50, yield and mapping rate of long reads are summarized in Table 2.

Adaptive sampling provided an approximate 2x unit increase in average depth coverage for the targeted regions (from 6.7x to 8.5x). Other quality metric statistics yielded results similar to those observed with shallow WGS (sWGS) (Table 2). The average time required for wf-human-variation workflow execution was found to be approximately 4 hour 30 minutes, assuming optimal availability for required resources (32 CPUs, 128 GB memory), which can further be improved by appropriately scaling compute resources. End-to-end sequencing analysis was completed within 4 days.

### Performance of ONT WGS Platform for Detection of Copy Number Changes

The standard log2 ratio estimated by QDNASeq was used to determine copy number changes. A log2 ratio of 0 indicates no copy number change, a log2 ratio of ~ 0.5 corresponds to a single copy gain, and a log2 ratio of 1 reflects a two-copy gain, with increasingly higher values indicating additional gains. Conversely, negative log2 ratios represent copy number losses.

**Figure 2:**
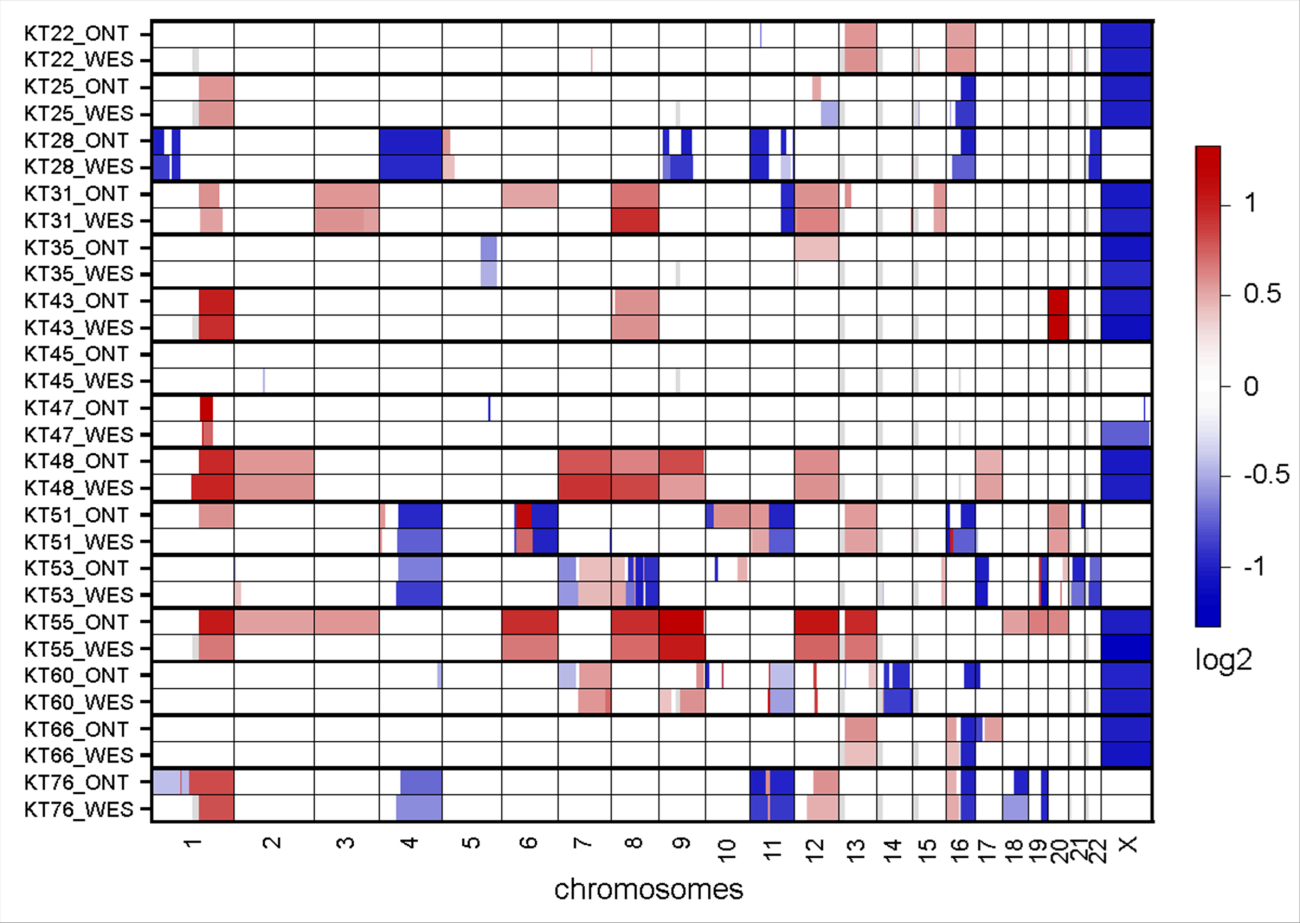
Heatmap summarizing results obtained from ONT WGS and WES/MLPA. Shown are the log2 scores of copy number changes observed in 15 xenograft samples using ONT sWGS and WES data.

**Figure 3:**
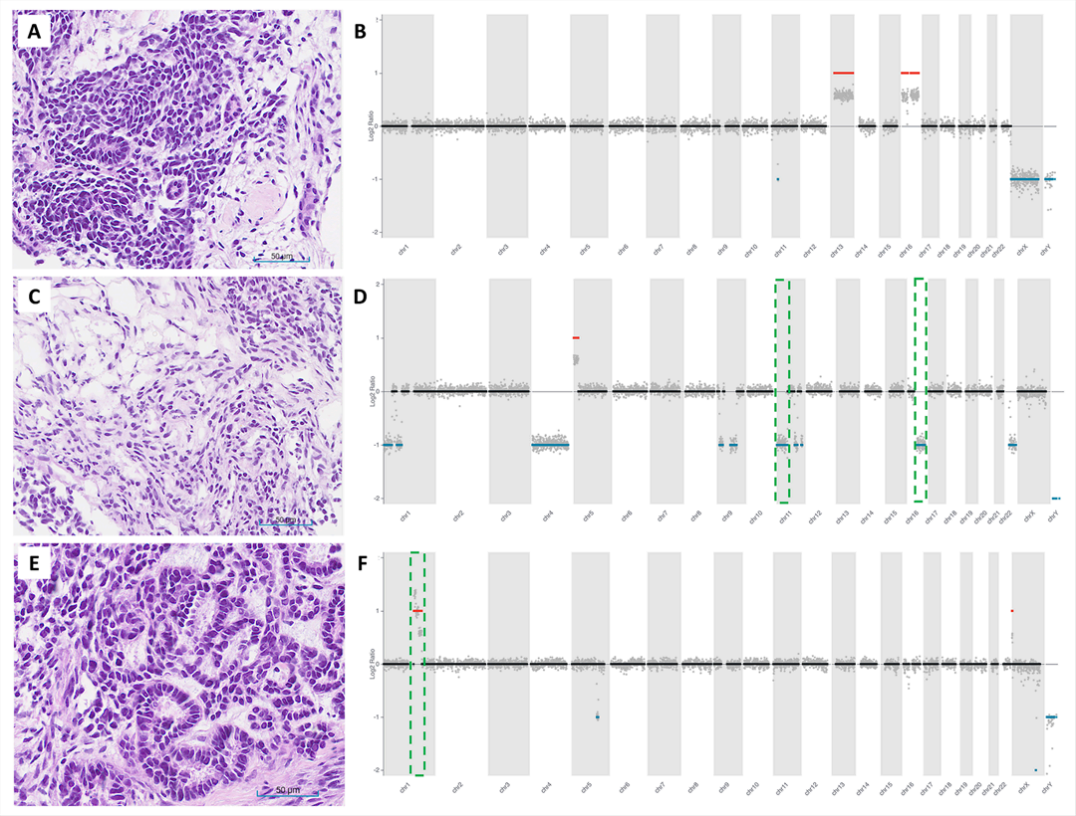
Composite image showing 3 ONT WGS CNV plots (samples KT-22, KT-28, and KT-47) side-by-side with H&E images from the corresponding xenografts. All three xenografts demonstrate morphological features of favorable histology Wilms tumors. KT-22 (**A)** displays triphasic morphology; KT-28 (**C**) is mainly composed of stromal elements, and KT-47 (**E**) exhibits early epithelium and blastemal components. Hematoxylin & Eosin, 200x. (**B**) ONT WGS did not identify any CNVs that are clinically significant in WT (i.e. 1p deletion, 1q gain, 11p15 deletion, and 16q deletion) in sample KT-22. (**D**) Deletions involving 11p and 16q (green, dashed line) were detected in KT-28. (**F**) 1q gain (green, dashed line) was found in sample KT-47 by the ONT WGS analysis. Both the WT relevant CNVs (green, dashed line) and the other CNV alterations in these samples—copy gains shown as red horizontal lines and deletions as blue horizontal lines—were confirmed by WES (Table 3).

sWGS detected 94% (16/17) of the CNVs that are clinically relevant in WT (Table 3, Figs. 2 and 3). In sample KT-53, the 16q deletion detected by MLPA^15^ was not observed with sWGS or WES. Intra-tumor heterogeneity and differences in test methodology between MLPA and sequencing may account for the discordant results. sWGS detected a 1q gain in sample KT-51 that was not identified by WES. Both sWGS and WES concordantly detected a 16q deletion in this sample. As anticipated, no WT-relevant CNVs were identified in samples KT-22, KT-35, and KT-45 by sWGS. We observed > 95% concordance between ONT sWGS-based approach and WES for the additional CNVs detected in this cohort (Fig. 2). Some of the notable discrepancies included the detection of a focal loss in chromosome 10p and gain of 10q in sample KT-51 by sWGS, which was not detected by WES. Likewise, focal 17p deletion and 17q gain were detected only by sWGS in sample KT-66. In addition, gain of chromosomes 2, 3, 18, 19, and 20 identified by sWGS in sample KT-55 was not confirmed by WES analysis (Fig. 2). Such discrepancies may be attributed to intra-tumor heterogeneity of the xenograft samples sequenced.

### Performance of ONT WGS Platform for Assessment of 11p15 Methylation Status

**Figure 4:**
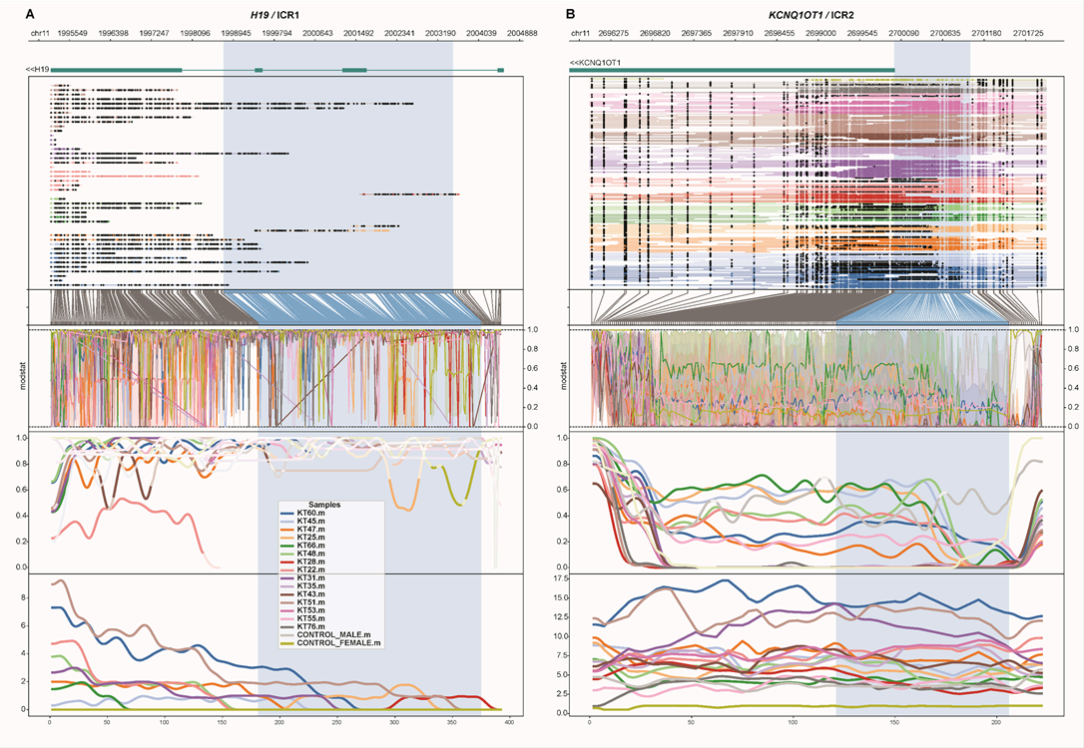
Methylation and coverage profiles of imprinting control regions (ICR) *H19*/ICR1 and *KCNQ1OT1*/ICR2. (**A**) Methylation profile around *H19*/ICR1 and (**B**) *KCNQ1OT1*/ICR2 generated using methylartist (see Methods). Blue shaded regions indicate the ICR region. The tracks from top to bottom indicate the following - genomic coordinates of the region (hg38), genes in the region, mapped reads (filled and open circles respectively show methylated and unmethylated CpG sites), raw base modification (methylation) scores (modstat), smoothened methylation scores and depth of coverage. The xenograft (n=15) and control samples (n=2) are shown in different colors as indicated in the legend. Adaptively sequenced samples were used wherever available.

Methylation profiles of all the xenograft samples in two imprinting control regions (ICRs) located at chromosome 11p15 were compared with those of two control samples (male and female, Human Genomic DNA, Promega, Fitchburg, WI) (Fig. 4). As per report from COG AREN0532, 30% to 70% methylation of both *H19/*ICR1 (chr11:1998744–2003508; hg38) and *KCNQ1OT1/*ICR2 (chr11:2699998–2700998; hg38) was considered to be retention of imprinting (ROI); 80% to 100% methylation of *H19*/ICR1 and 30% to 70% methylation of *KCNQ1OT1/*ICR2 was considered to be LOI; and 80% to 100% methylation of *H19* and 0% to 20% methylation of *KCNQ1OT1/*ICR2 was considered to be loss of heterozygosity (LOH)^6^. All the xenograft samples showed expected methylation levels in the *KCNQ1OT1/*ICR2 region; however, the *H19*/ICR1 region did not show sufficient depth of coverage to determine methylation level (Fig. 4). Six WT xenograft samples (KT-25, KT-45, KT-47, KT-48, KT-60 and KT-66) previously reported by MLPA analysis to have undergone LOI showed 30% to 70% methylation of *KCNQ1OT1/*ICR2 (green lines in Figure S1). In contrast, samples previously characterized as having copy neutral LOH (KT-31, KT-35, KT-43, KT-51 and KT-53) and loss of maternal copy of chromosome 11p15 (KT-76 and KT-28) showed ~ 0% methylation in this region (magenta and orange lines in Figure S1 respectively). The six samples with LOI (n = 6) showed a statistically significant gain in methylation of *KCNQ1OT1/*ICR2 when compared with the xenograft samples with copy neutral LOH (n = 5; two-sided Mann-Whitney U test *P* = 0.04; Figure S2). The overall sequencing coverage of control samples (< 5x) was not adequate for this comparison. In addition, due to low sequencing coverage at the ICR1 locus, the methylation level in *H19/*ICR1 could not be determined by this analysis (Fig. 4A).

### Performance of ONT Adaptive Sampling for Detection of Copy Number Changes

Results from adaptive sampling and sWGS were 100% concordant for the clinically relevant copy number changes in WT (i.e., 1p deletion, 1q gain, 11p15 deletion, and 16q deletion) (Table 3 and Figure S3). For all other CNVs, slight discordance was observed only in a chromosome 11q segment of sample KT-60, where the size of the 11q deletion was increased by ~20 Mbp after adaptive sampling (from chr11: 80,000,001–135,086,622 to chr11: 60,000,001–135,086,622) with a decrease in log2 ratio from −0.97 to −0.47. Such discrepancies may be explained by the increased sequencing depth and higher analytical sensitivity of the sequencing analysis using adaptive sampling, whereby the neighboring regions of 11q that did not get sufficiently sequenced by sWGS but were adequately covered after adaptive sampling. These results overall indicate high reproducibility of CNV calls even at shallow depth of sequencing before and after adaptive sampling, suggesting that adaptive sampling can play an important role in determining clinically relevant copy number changes (Figure S3).

### Performance of ONT Adaptive Sampling for Assessment of 11p15 Methylation Status

The trend of methylation profiles in ICR2 region overall remained the same after adaptive sampling with an expected increase in the number of methylated CpG sites for quantification. The ICR1 regions remained under covered, precluding conclusive results (Figure S4).

## DISCUSSION

This is the first study to evaluate the performance of sWGS and adaptive sampling using nanopore technology for the detection of CNVs and 11p15 LOI in WT. Molecular assessment of LOH at chromosomes 1p and/or 16q has been incorporated into COG risk stratification for favorable-histology WT based on prospective findings from NWTS-5^3^ and subsequent validation in AREN0532 and AREN0533^22^. In NWTS-5 (> 1,700 patients), LOH at 1p and/or 16q was an independent predictor of relapse and mortality, with the highest risk observed in patients with WT harboring combined 1p/16q LOH. Across both early- and advanced-stage cohorts, patients with WT having 1p/16q LOH had significantly inferior relapse-free survival despite standard therapy, supporting systemic treatment intensification^3^. Building on this, AREN0532 and AREN0533 demonstrated that biomarker-directed augmentation of chemotherapy improved event-free survival^22^. Although LOI of 11p15 is also considered to be a prognostic indicator in stage I WT in young children, it is not incorporated into the ongoing trial study AREN2231 because current testing technologies are not routinely available and do not return results fast enough to be used in upfront clinical decisions at this time^7^. Accurate LOI assessment requires allele-specific methylation resolution, which conventional bisulfite sequencing and methylation arrays are generally unable to provide. Reliable detection of allele-specific methylation necessitates simultaneous profiling of both allele-informative SNPs and DNA methylation marks within the same or directly linked sequencing data. To this end, long-read nanopore sequencing provides direct single-molecule resolution for detection of cytosine modifications in native DNA and because reads span kilobases, enables haplotype-aware, allele-specific methylation analyses required to detect LOI. In addition, compared with traditional short-read sequencing and microarray-based methods, nanopore-based sequencing offers a substantially faster turnaround, delivering results in approximately 5 days instead of the 2–4 weeks typically required by conventional approaches. Current favorable histology WT therapies start with a cycle of vincristine and actinomycin-D to allow for results that affect final risk stratification to return. In the future, shorter turnaround time for molecular results could allow more intense or targeted therapies to be delivered earlier for high-risk groups. Also, diagnostic conundrums regarding the presence of diffuse or focal anaplasia could be informed by earlier reporting of molecular results at the 17p13 (*TP53*) locus. These capabilities could facilitate a timelier and more comprehensive stratification of patients with favorable histology WT for risk-adapted first-line treatment, making nanopore an attractive option for WT testing.

To evaluate the feasibility of CNV and 11p15 LOI detection using nanopore, we tested 15 WT xenograft samples that were previously characterized by WES and MLPA. We opted to use the MinIon platform for this pilot study due to its affordability and ease of use, which could facilitate deployment for single sample testing in our center, as well as its potential application in under-resourced settings. Our results indicate that MinIon sequencing can perform on par with conventional short-read WES for CNVs that are clinically relevant in WT. In our cohort, we detected all WT-relevant CNVs except for one alteration (16q deletion) in one xenograft sample (KT-53). This tumor was found to have diffuse anaplasia, and thus 16q deletion would not have been clinically actionable in this specific case. The analysis did not yield any false positive calls. Nonetheless, the MinION platform has limitations that should be recognized, including its relatively low sequencing throughput (~ 20 Gb per flow cell) compared with other nanopore sequencers (e.g., PromethION, ~ 100–300 Gb per flow cell) and short-read platforms, which limits its achievable sequencing depth. In our study, the read depth obtained by MinION sequencing, even with adaptive sampling, was insufficient to enable effective assessment of methylation status at 11p15, particularly for the *H19*/*ICR1* locus (Fig. 4). Despite the limitation, the WT relevant CNV data obtained by MinION sequencing are comparable in scope to those produced by COG-recommended SNP array testing.

## CONCLUSIONS

In conclusion, this study demonstrates the feasibility of using nanopore sequencing for the assessment of clinically relevant CNVs in WT, and highlights the potential of this technology for the integrated assessment of CNV and 11p15 methylation status in WT. Future work will focus on validating this approach in a prospective patient cohort with the aim of developing a clinical-grade assay capable of providing rapid, targeted detection of clinically actionable chromosomal and epigenetic alterations to inform clinical decision-making. Fresh tumor samples obtained directly at the time of the procedure (nephrectomies or biopsies) represent an optimal substrate for nanopore sequencing because high-quality DNA can be extracted without the degradation or artifacts introduced by formalin fixation or extended storage. Because nanopore library preparation relies on streamlined workflows, tissue collected in the operating room can be transitioned directly to sequencing with minimal processing, allowing timely results and facilitating integration into clinical testing workflows. The current findings underscore that successful clinical integration will require higher-capacity sequencing platforms, with the PromethION representing a particularly suitable option due to its scalability, throughput, and reduced computational demands.

## Supplementary Material

Supplementary Files

This is a list of supplementary files associated with this preprint. Click to download.

• Table1.docx

• Table2.xlsx

• Table3.docx

• FigureS1.tif

• FigureS2.tif

• FigureS3.tif

• FigureS4.tif

• SupplementalTable1.xlsx

Tables

Tables are available in the Supplementary Files section

## Data Availability

The data that support the findings of this study are available from the corresponding author upon reasonable request.

## Abbreviations

BED: Browser Extensible Data
CNV: Copy number alteration
COG: Children’s Oncology Group
ICR: Imprinting Control Regions
LOH: Loss of heterozygosity
LOI: Loss of imprinting
MLPA: Multiplex ligation-dependent probe amplification
ONT: Oxford Nanopore Technologies
PDX: Patient-derived xenograft
sWGS: Shallow whole genome sequencing
SNP: Single nucleotide polymorphism
WES: Whole exome sequencing
WGS: Whole genome sequencing
WT: Wilms tumor
